# Childhood Sexual Abuse, Sexual Behavior, and Revictimization in Adolescence and Youth: A Mini Review

**DOI:** 10.3389/fpsyg.2019.02018

**Published:** 2019-08-30

**Authors:** Ángel Castro, Javier Ibáñez, Berta Maté, Jessica Esteban, Juan Ramón Barrada

**Affiliations:** Department of Psychology and Sociology, University of Zaragoza, Teruel, Spain

**Keywords:** childhood sexual abuse, sexual risk behaviors, revictimization, adolescents and young adults, traumagenic dynamics model, information-motivation-behavioral skills model

## Abstract

Childhood sexual abuse (CSA) is considered as an activity aimed at providing sexual pleasure, stimulation, or sexual gratification to an adult who uses a minor for this purpose, taking advantage of the situation of superiority. CSA can have devastating consequences for the child. Previous studies have concluded that those who suffer an episode of CSA perform more risky sexual behaviors and are more likely to experience further episodes of sexual victimization during adolescence and early youth. There are two theoretical contributions that, although they offer partial views, can help to understand the association between CSA, sexual behavior, and revictimization in adulthood: the traumagenic dynamics model and the information-motivation-behavioral skills model. This short review provides an overview of the problems and theoretical explanations that have been presented up to the present, underlining the importance of prevention and sex education as of childhood, as well as the need to continue investigating in order to develop specific theoretical models that help to understand and prevent CSA and its consequences.

## Introduction

The objective of this short review is to offer a brief overview of childhood sexual abuse (CSA) and its consequences in the field of sexuality, paying special attention to the performance of risky sexual behaviors and sexual revictimization in adolescence and early youth. In addition, two theoretical explanations that address—albeit partially—the relationship between CSA, risky sexual behavior, and sexual revictimization are highlighted. That is the structure that is followed in the text. Finally, conclusions are drawn, underscoring the need to continue investigating this phenomenon, developing specific theoretical models and designing and implementing prevention programs for CSA.

## Child Sexual Abuse

In many cases, child sexual abuse has devastating consequences for the lives of those who suffer it, as it involves the destructuring of the child’s behavior and emotions and, sometimes, serious interference in his or her development (Clayton et al., 2018). CSA is considered a serious health and social problem in every country in the world. It can be defined as the activity in which an adult, taking advantage of his or her superiority, uses a minor to provide sexual pleasure, stimulation, or sexual gratification (Sánchez-Meca et al., 2011). CSA may occur through physical contact (e.g., touching, vaginal, oral, or anal sex, both perpetrated and suggested), or by viewing pornography, adult exhibition, or requests for sexual favors (Finkelhor, 1979).

Reviews carried out in recent years on the subject report prevalence rates ranging from 5 to 18% of minors, depending on the geographical and cultural context in which the studies are conducted (Senn et al., 2017; Clayton et al., 2018; Gray and Rarick, 2018). Because of the magnitude of these rates, some authors claim that CSA is a public health problem that is far from being solved (Gray and Rarick, 2018).

As for the abusers, in about 85% of cases, they are male and their age is usually between 30 and 40 years (Pereda et al., 2016; Clayton et al., 2018; Gray and Rarick, 2018). Abusers, especially in the case of girls, usually belong to the child’s closest environment, and are people she loves (Pereda et al., 2016). The places where episodes of abuse occur are often the most frequented by the children, such as their homes, schools, or leisure centers. Unlike other types of abuse, in CSA, the economic level, either of the abuser or of the victim, has no influence (Senn et al., 2012). With regard to the victims, Senn et al. (2017) state that the maximum risk age range is between 6 and 12 years, and that girls are more than three times as likely as boys to be abused. However, it is noted that, due to aspects related to masculinity and fear of being labeled and stigmatized, boys often do not admit having been abused, so the proportion of abused boys could be higher (Homma et al., 2012; Pereda et al., 2016).

For several decades, some, studies have reported that about one-third of male abusers may have been a victim in their childhood (Finkelhor, 1979; Senn et al., 2012). Therefore, although it cannot be stated that there is intergenerational transmission of abuse, because the majority of victims are female and they do not subsequently become abusers, it is relatively common for abusers to have witnessed or suffered abuse during their childhood (Clayton et al., 2018).

## The Consequences of Child Sexual Abuse

Having suffered some episode of CSA has been linked to poorer psychological functioning (Senn et al., 2008; Homma et al., 2012), aggressive behavior, interpersonal problems, educational difficulties, or increased use of alcohol and other drugs (Clark et al., 2007). CSA is a violation of the child’s privacy, which can lead to distrust of others. Therefore, one of the main areas of study and intervention in the consequences of CSA is that of interpersonal relationships and sexuality. In the victims of CSA, incongruous, and even contradictory behaviors are observed in the area of sexuality: either they avoid relating to others for fear of possible revictimization (Homma et al., 2012) or the opposite occurs—they suffer alterations in sexuality that lead to the performance of risky sexual behaviors (Senn et al., 2008).

### Risky Sexual Behaviors

CSA has been associated with the performance of risky sexual behaviors in adolescence and youth (Senn et al., 2008). Adolescence is a critical period, in which sexual activity begins and sexual behavior is subject to a multitude of influences (e.g., friends, media, pornography) that can promote a decrease in the control of the situation and increased vulnerability (Spanish Foundation for AIDS Research and Prevention, 2003). Some characteristic aspects of sexuality during adolescence may include low risk perception (Castro and Santos-Iglesias, 2016), alcohol and other drug use, lack of planning of sexual intercourse, the romantic ideals characteristic of this stage (Jones and Furman, 2011), scarce eroticization of condom use (DiClemente et al., 2004), or poor skills to negotiate condom use and perform safe sexual behaviors (Santos-Iglesias and Sierra, 2012).

Vulnerability is even greater if, to these obstacles to engage in protective sexual behavior that exist during adolescence, is added the fact that the adolescent has been a victim of CSA. Some research concludes that people who have suffered an episode of CSA present earlier initiation of consensual sex with penetration (Thornton and Veenema, 2015; Gray and Rarick, 2018), more sexual partners (Senn et al., 2012, 2017; Walsh et al., 2013), more inconsistent condom use (Senn et al., 2012, 2017), or increased drug use in sexual relationships (Thornton and Veenema, 2015). All these behaviors imply greater vulnerability to sexually transmitted infections (STI; Homma et al., 2012; Senn et al., 2012) and are usually variables that also predispose one to suffer new episodes of sexual victimization (Bermúdez et al., 2010).

### Sexual Revictimization

Suffering further episodes of sexual victimization during adolescence and early youth is common among victims of CSA. Authors such as Walker et al. (2017) concluded in their meta-analysis that the prevalence of revictimization reached almost 50% of the cases. Some studies have reported that female victims of CSA are three to five times more likely to suffer further sexual assault than those who have not suffered CSA (Pereda et al., 2016; Godbout et al., 2019).

Some variables have been proposed in the existing literature to explain the CSA-sexual assaults relationship in adulthood (Walker et al., 2017). For example, Santos-Iglesias and Sierra (2012) suggest that this relationship is mediated by three variables: sexual experience, sexual assertiveness, and substance use before intercourse. In terms of sexual experience, it has been established that women who suffered CSA have a larger number of partners, which increases the risk of revictimization due to a probabilistic issue: the more partners, the more likely they are to be aggressive (Arata, 2000).

Regarding sexual assertiveness, authors like Livingston et al. (2007) claim that minors suffering CSA are socialized in a victim role, which makes them less capable of being assertive to reject others’ sexual advances, which influences a possible revictimization. Substance consumption may also mediate this relationship, as these authors have indicated that CSA is a risk factor for increased consumption and this, in turn, is a risk factor for revictimization.

## Theoretical Models: Traumatogenic Dynamics and Information-Motivation-Behavioral Skills

The relationship between having suffered an episode of CSA and the subsequent performance of risky sexual behaviors and suffering new cases of victimization is well established in the extant literature. However, there is little empirical research on how this relationship occurs (Senn et al., 2012), and there are no specific theoretical models to explain it. There are two theoretical contributions that, although understudied so far and providing partial views, may help to understand the relationship between CSA, risky sexual behavior, and revictimization in adulthood. The traumagenic dynamics model (TD; Finkelhor and Browne, 1985) focuses on CSA and its consequences in the field of sexuality. For its part, the information-motivation-behavioral skills model (IMB; Fisher and Fisher, 1992) focuses on the performance of risky sexual behaviors.

### Traumatogenic Dynamics Model

According to this model, CSA can have four negative consequences. First, a traumatic sexualization (Matorin and Lynn, 1998), through which incorrect sexual behavior scripts are developed because negative sexual patterns are rewarded. This may motivate individuals who were sexually abused to have a large number of sexual partners, to wish to engage in risky behaviors, or to have sex in exchange for material rewards (Senn et al., 2012; Walsh et al., 2013). Second, a sense of betrayal (DiLillo and Long, 1999), as the child feels deceived by the abuser or by the reactions of others upon finding out about the abuse. This may be related to the difficulty of trusting others, which, in turn, may influence rejecting stable relationships in favor of multiple and sporadic relationships, with their entailed risk both of performing risky sexual behaviors and possible episodes of sexual victimization (Senn et al., 2008, 2017).

The third element of the model is stigmatization, as the individual feels sexually different, in addition to feeling shame and guilt (Feiring et al., 2001). These feelings can motivate abused people to internalize their role and have multiple partners, as well as to carry out risky behaviors (Senn et al., 2008). Fourth and last, Finkelhor and Browne (1985) refer to the loss of power in their relationships, as they feel that they cannot control their sexual interactions and are incapable of rejecting sex or risky relationships (Gwandure, 2007).

There is evidence that CSA is related to the constructs of the traumatogenic dynamics model (Senn et al., 2012). In addition, the four constructs of the model have been associated with poorer psychological functioning. Higher traumatic sexualization was associated with more anxiety and sexual avoidance and with lower sexual self-esteem (Hazzard, 1993; Matorin and Lynn, 1998); increased feeling of betrayal was associated with more interpersonal problems (Hazzard, 1993); and more stigmatization and powerlessness have been linked to less sexual self-esteem and more distress and depression (Hazzard, 1993). Thus, the TD model is shown to provide an explanation of the consequences of CSA, both psychological and sexual, which can influence the increased performance of risky sexual behaviors and sexual revictimization in adolescence and youth (Walsh et al., 2013).

### Model of Information-Motivation and Behavioral Skills

Fisher and Fisher (1992) developed this theoretical model to explain sexual behavior based on three elements, which can serve to explain the relationship between CSA and the performance of risky sexual behaviors. They suggest that information (on the transmission, prevention, and consequences of STI), motivation (to protect oneself, about safe sex), and behavioral skills (to discuss safe sex with a partner and to use condoms) influence sexual behavior, as several studies have shown (Johnson et al., 2006; Fisher et al., 2014; Jones et al., 2018; Ybarra et al., 2018).

CSA can influence the level of information, motivation, and behavioral skills for safe sex through several mechanisms. In fact, suffering CSA has been linked to less knowledge about STI, fewer favorable attitudes toward safe sex, and fewer behavioral skills and lower self-efficacy (Hall et al., 2008).

Zurbriggen and Freyd (2004) argue that CSA may cause dissociative tendencies, which could interfere with the coding and processing of sexuality-related information. Thus, people who were sexually abused during childhood may have difficulty processing or remembering information about sex, which can lead to gaps in their knowledge about prevention and about risky behaviors.

Similarly, CSA can lead to difficulties in distinguishing fantasy from reality, because many abusers lie or distort reality. Being motivated to have safe sexual practices may require understanding that one is at risk of an STI; if individuals are not capable of objectively assessing the reality of sexual situations, they will not believe that they are vulnerable. Thus, the low or lack of perception of risk can lead to a low or lack of motivation to practice safe sex (Zurbriggen and Freyd, 2004). Finally, individuals who were sexually abused as children may have learned that they cannot control what happens to them, which can lead to low self-efficacy and few skills to negotiate safe sex (Zurbriggen and Freyd, 2004).

## Conclusions

CSA currently remains a serious public health problem in all countries worldwide, as prevalence data confirm. Therefore, the objective of this short review was to present a summary of its definition, characteristics, consequences in the area of sexuality and of the theoretical approaches that attempt to explain—albeit partially—the relationship between CSA and the performance of risky sexual behaviors and sexual revictimization in adolescence and youth.

As in most studies that focus on the sexuality of children, adolescents, and young people, we must demand more and better education in this area, from an early age and without taboos (Castro and Santos-Iglesias, 2016). The lack of information and normalization of these topics in the habitual conversations between minors and adults, coupled with the low and/or poor sexual education provided in schools, places children in a situation of ignorance and vulnerability for the prevention and recognition of risky situations, such as CSA (Senn et al., 2008).

The focus should therefore be on the importance of prevention and promotion of sexual health, teaching from early ages how to identify possible situations or intentions of abuse, educating in gender equality, and promoting healthy sexuality (Castro and Santos-Iglesias, 2016). This is the main way to try to reduce this problem, starting with the basics.

From a scientific viewpoint, it is necessary to continue research on the subject, not only empirically, but also creating specific theoretical models that can explain the effects of CSA and how it influences the victims’ sexuality (Senn et al., 2008). Although they may serve to explain certain aspects of this relationship, in this review, we have seen how the proposed theoretical models only provide partial views (Senn et al., 2012). Hence, there is a long way to go, but the severity of the topic and the terrible consequences that CSA usually has on children’s lives make up for the efforts required.

## Author Contributions

JI, BM, and JE did bibliographic search and wrote the first draft of the manuscript. ÁC and JB contributed to the conception and design of the study, reviewed the first draft of the manuscript, and wrote the final version of the manuscript. All authors contributed to manuscript review, read and approved the submitted version.

### Conflict of Interest Statement

The authors declare that the research was conducted in the absence of any commercial or financial relationships that could be construed as a potential conflict of interest.
